# Usability of the Video Head Impulse Test: Lessons From the Population-Based Prospective KORA Study

**DOI:** 10.3389/fneur.2018.00659

**Published:** 2018-08-17

**Authors:** Maria Heuberger, Eva Grill, Murat Saǧlam, Cecilia Ramaioli, Martin Müller, Ralf Strobl, Rolf Holle, Annette Peters, Erich Schneider, Nadine Lehnen

**Affiliations:** ^1^Department of Neurology, University Hospital Munich, Ludwig-Maximilians Universität München, Munich, Germany; ^2^German Center for Vertigo and Balance Disorders, University Hospital Munich, Ludwig-Maximilians Universität München, Munich, Germany; ^3^Faculty of Medicine, Institute for Medical Information Processing, Biometry and Epidemiology, Ludwig-Maximilians Universität München, Munich, Germany; ^4^Munich Center of Health Sciences, Ludwig-Maximilians Universität München, Munich, Germany; ^5^Institute of Medical Technology, Brandenburg University of Technology, Cottbus-Senftenberg, Germany; ^6^Faculty of Applied Health and Social Sciences, Rosenheim University of Applied Sciences, Rosenheim, Germany; ^7^Institute of Health Economics and Health Care Management, Helmholtz Zentrum München, German Research Center for Environmental Health, Neuherberg, Germany; ^8^Institute of Epidemiology, Helmholtz Zentrum München, German Research Center for Environmental Health, Neuherberg, Germany; ^9^Department of Psychosomatic Medicine and Psychotherapy, Klinikum Rechts der Isar, Technical University of Munich, Munich, Germany

**Keywords:** video head impulse test, vHIT, saccades, vestibulo-ocular reflex, VOR, artifact, technical mistake

## Abstract

**Objective:** The video head impulse test (vHIT) has become a common examination in the work-up for dizziness and vertigo. However, recent studies suggest a number of pitfalls, which seem to reduce vHIT usability. Within the framework of a population-based prospective study with naïve examiners, we investigated the relevance of previously described technical mistakes in vHIT testing, and the effect of experience and training.

**Methods:** Data originates from the KORA (Cooperative Health Research in the Region of Augsburg) FF4 study, the second follow-up of the KORA S4 population-based health survey. 681 participants were selected in a case-control design. Three examiners without any prior experience were trained in video head impulse testing. VHIT quality was assessed weekly by an experienced neuro-otologist. Restrictive mistakes (insufficient technical quality restricting interpretation) were noted. Based on these results, examiners received further individual training.

**Results:** Twenty-two of the 681 vHITs (3.2%) were not interpretable due to restrictive mistakes. Restrictive mistakes could be grouped into four categories: slippage, i.e., goggle movement relative to the head (63.6%), calibration problems (18.2%), noise (13.6%), and low velocity of the head impulse (4.6%). The overall rate of restrictive mistakes decreased significantly during the study (12% / examiner within the first 25 tested participants and 2.1% during the rest of the examinations, *p* < 0.0001).

**Conclusion:** Few categories suffice to explain restrictive mistakes in vHIT testing. With slippage being most important, trainers should emphasize the importance of tight goggles. Experience and training seem to be effective in improving vHIT quality, leading to high usability.

## Introduction

The video head impulse test (vHIT) is highly popular in the work-up for dizziness and vertigo. In neuro-otology settings, it is considered the primary investigation to determine vestibular hypofunction (1, 2). Being increasingly used in emergency departments to distinguish stroke from peripheral vestibular problems (3, 4), its handling and interpretation becomes important also for non-specialists.

The vHIT tests the high frequency vestibulo-ocular reflex (VOR), which stabilizes gaze during passive head movement (5, 6). In contrast to the clinical head impulse test (7), the vHIT is a quantitative test, able to detect covert re-fixation saccades that appear during the head movement (8). This leads to a sensitivity and specificity comparable to the gold standard for quantitative head impulse testing, the search-coil-in-magnetic-field-technique (6, 9). In contrast to the search-coil-in-magnetic-field-technique, the vHIT is non-invasive, mobile, and quick.

However, recent studies suggest a number of pitfalls, which seem to be challenging vHIT usability (10–13). In addition to problems like low head stimulus velocity preventing the detection of slight deficits (14), a number of different types of artifacts have been identified (9, 10, 12, 13, 15, 16). It is not clear how relevant each of these problems is for vHIT interpretability, how often they occur in naïve examiners and how they are influenced by experience and training. Here, within the framework of the population-based Cooperative Health Research in the Region of Augsburg (KORA) survey with 681 participants and non-specialist examiners, we identify which technical problems are relevant in vHIT testing and how they are affected by experience and training.

## Material and methods

### Participants

Data originates from the KORA (Cooperative Health Research in the Region of Augsburg) FF4 study, the second follow-up of the KORA S4 population-based health survey. 2,279 subjects participated in the FF4 study (from 03.06.2013 to 27.09.2014, mean age was 60.8 years ranging from 39 to 88 years, 51.6% female). Participants reporting at least moderate vertigo or dizziness within the last 12 months in a face-to-face interview (570 participants) were intended to be evaluated with vHIT on the same day. A random sample of 233 asymptomatic participants, representative for the study population, were planned as controls. Twenty participants were part of a pilot feasibility study with one examiner. 142 participants had to be excluded because of problems of the cervical spine, e.g., an acute cervical disc herniation or spinal fracture. In total, vHIT data of 681 participants were assessed.

### Ethics statement

The study was carried out in accordance with the Declaration of Helsinki, including written informed consent of all participants. All study methods were approved by the ethics committee of the Bavarian Chamber of Physicians, Munich (FF4: EC No. 06068).

### Video head impulse testing (vHIT)

**Figure 1:**
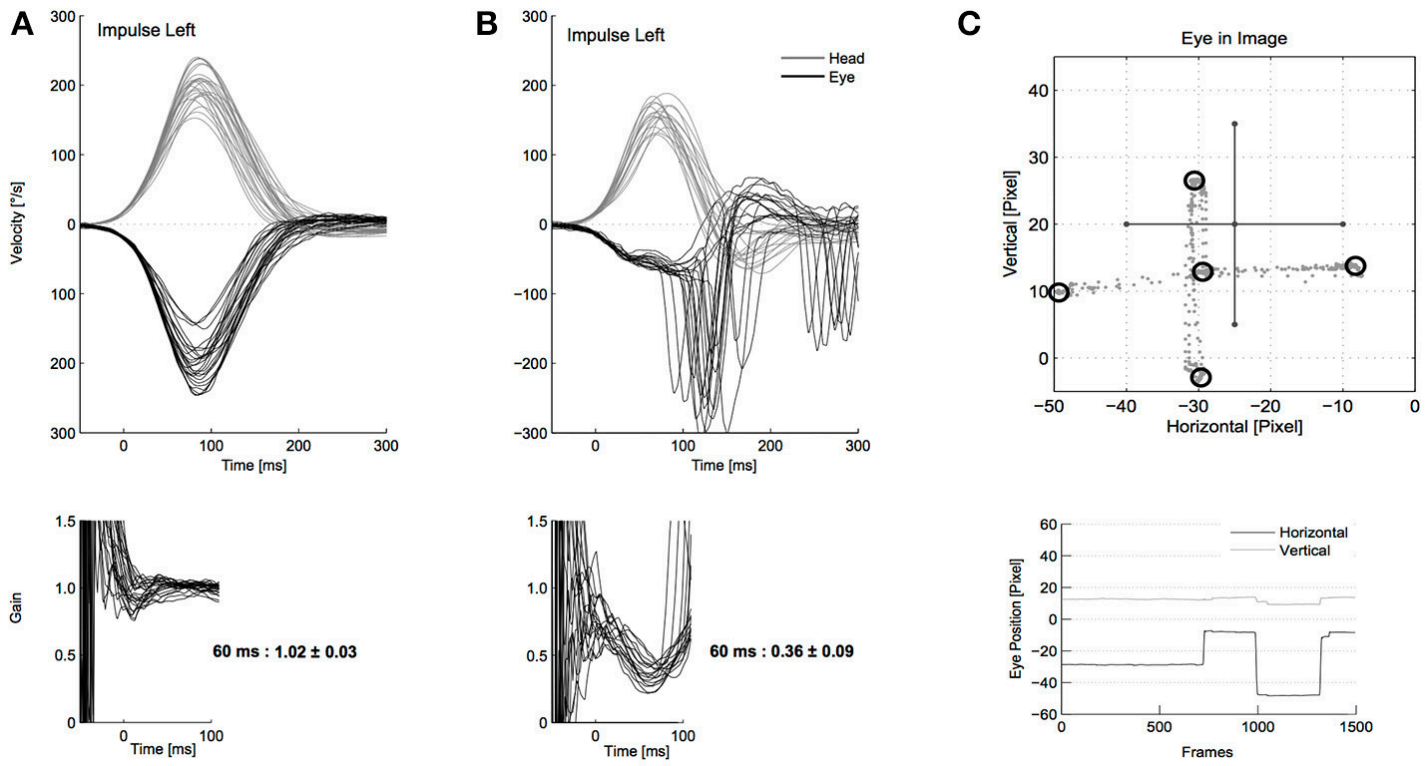
Video head impulse test (vHIT) examples with good technical quality. **(A)** Shows a normal video head impulse test (vHIT) with the eye movement (black, top) matching the head movement (gray, top), resulting in a gain at 55–65 ms around 1 (bottom). Head velocity is sufficient (around 150–250°/s) and there are no signs of restrictive mistakes. **(B)** Shows a vestibular deficit with reduced gain, covert, and overt re-fixation saccades. **(C)** Shows a successful calibration with the eye movements (gray dots, top) forming a cross, accumulating in the five laser points (black circles, top). In the bottom of **(C)** some original eye movement recordings during calibration are shown.

Standard vHIT was performed using the EyeSeeCam system [(5), procedure as described in (17) and in Supplement 1, examples with good technical quality see Figure 1]. The participant was seated two meters from a wall with a fixation point at eye level. The examiner applied horizontal head impulses from behind the participant via the jaw (25° head-down position, targeted velocity 150–250°/s, amplitude 6–12°, 10–15 head impulses to each side).

### Examiners and training

Three examiners without any prior experience in head impulse testing were trained in performing the vHIT according to the standard operating procedure (Supplement 1, examiners' professions: graduated nurse, doctor's assistant, chemical technical assistant, age at begin of testing: 48, 60, and 61 years, all female). VHITs were done for 16 months, including training and the pilot study of 1 month. Only examiner 1 participated in a first training of 2 h and the pilot study with 20 participants. Examiners 1–3 received two sessions of joint, individually adjusted, training for two to 3 h before data acquisition started. During data acquisition vHITs were analyzed regarding technical quality on a weekly basis by an experienced neuro-otologist. Based on the evaluation results, examiners received two further joint, individually adjusted, trainings (month 2 and 4, 2 h each). After the fourth month no further training was necessary for the remaining 12 months.

Examiner 1 assessed 261 participants (including the pilot study), examiner 2,219 participants and examiner 3,200 participants. In one participant with sufficient vHIT quality the examiner was not noted.

### Mistake analysis

**Figure 2:**
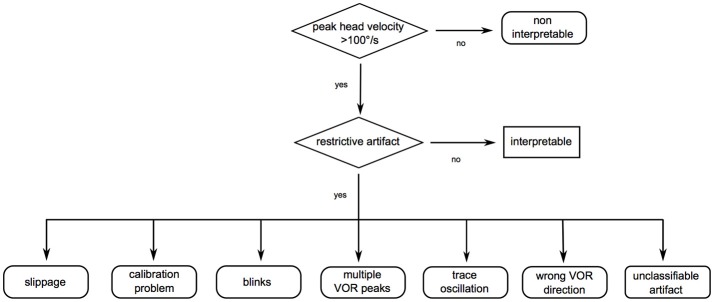
Flowchart to scan for restrictive mistakes. Restrictive mistakes are artifacts preventing a confident overall video head impulse test (vHIT) interpretation of one participant (restrictive artifacts) or an insufficient head velocity stimulus in all vHIT traces of one participant. First, sufficient peak head velocity was checked (>100°/s). In a second step, data were scanned for artifacts possibly restricting overall interpretability [in analogy to (10)]: (1) Slippage: phase shift of the head with respect to eye motion, probably due to a loose goggle strap, the examiner touching the goggle strap while applying the head impulse or stretching the patient's skin. (2) Calibration problems: mostly due to participants not following the calibration instructions. Signs pointing to an insufficient calibration may be inappropriately high (>1.3) or low (< 0.79) vestibulo-ocular reflex (VOR) gain values without consecutive re-fixation saccades. (3) Blinks: oscillations crossing the baseline (no saccades, ≥75% of peak eye velocity). (4) Multiple VOR peaks: two or more eye velocity peaks during head movement (no saccades, ≥25% of peak eye velocity), probably due to the examiner touching the goggles, mini-blinks or impaired pupil detection. (5) Trace oscillations: oscillations (no saccades, < 25% of peak eye velocity during head movement and < 75% of peak eye velocity after head movement), probably due to impaired pupil detection. (6) Wrong VOR direction: no eye movement or eye movement in the same direction as the head movement, probably due to patient inattention. (7) Unclassifiable artifact: any other artifact, which restricts interpretation, but does not match the criteria above.

VHIT data were analyzed offline using a flowchart (Figure 2) to scan for restrictive mistakes. Mistakes were considered as restrictive when they prevented a confident overall interpretation of the vHIT per participant. This could be because of lack of vHIT traces with a sufficient head stimulus (peak velocity >100°/s) or because of lack of vHIT traces without artifacts masking or modifying the time frame for calculating the gain and/or the remaining eye movement trace for evaluating re-fixation saccades. The minimum number of traces for a confident interpretation was set to three. Artifacts were considered in analogy to (10).

Considered artifacts included:

1. Slippage: phase shift of the head with respect to eye motion. This can be due to a loose goggle strap resulting in head motion preceding eye motion. Another reason can be the examiner touching the goggle strap while applying the head impulse or stretching the patient's skin causing eye preceding head motion. Slippage can lead to abnormal VOR gain values without other signs of a deficit or normal gain values in the presence of relevant re-fixation saccades hiding a deficit.
2. Calibration problems: mostly due to participants not following the calibration instructions. Signs pointing to an insufficient calibration may be inappropriately high (>1.3) or low (<0.79) VOR gain values without consecutive re-fixation saccades.
3. Blinks: oscillations crossing the baseline (which do not qualify as saccades), ≥75% of peak eye velocity.
4. Multiple VOR peaks: Two or more eye velocity peaks during head movement (which do not qualify as saccades), ≥25% of peak eye velocity, inhibiting a secure gain calculation, probably due to the examiner touching the goggles, mini-blinks or impaired pupil detection (e.g., narrow palpebral fissure or participant wearing mascara).
5. Trace oscillations: oscillations (which do not qualify as saccades), <25% of peak eye velocity during head movement and <75% of peak eye velocity after head movement, inhibiting the evaluation of possible saccades/valuable gain calculation, probably due to impaired pupil detection.
6. Wrong VOR direction: no eye movement or eye movement in the same direction as the head movement, probably due to patient inattention.
7. Unclassifiable artifact: any other artifact, which restricts interpretation, but does not match the criteria above.

Data were scored regarding interpretability by two experienced neuro-otologists (MH, >5 years of experience, NL, >10 years of experience). If multiple restrictive mistakes were present in one participant's vHIT, the most relevant was noted.

### Statistical analysis

We report frequencies and relative frequencies for categorical data and mean and standard deviation for continuous data. To test differences of success probabilities we used a Chi-Square test. R 3.3.2 was used for all analyses (R Core Team 2014). Statistical significance was set at a two-tailed 5 % level.

## Results

**Figure 3:**
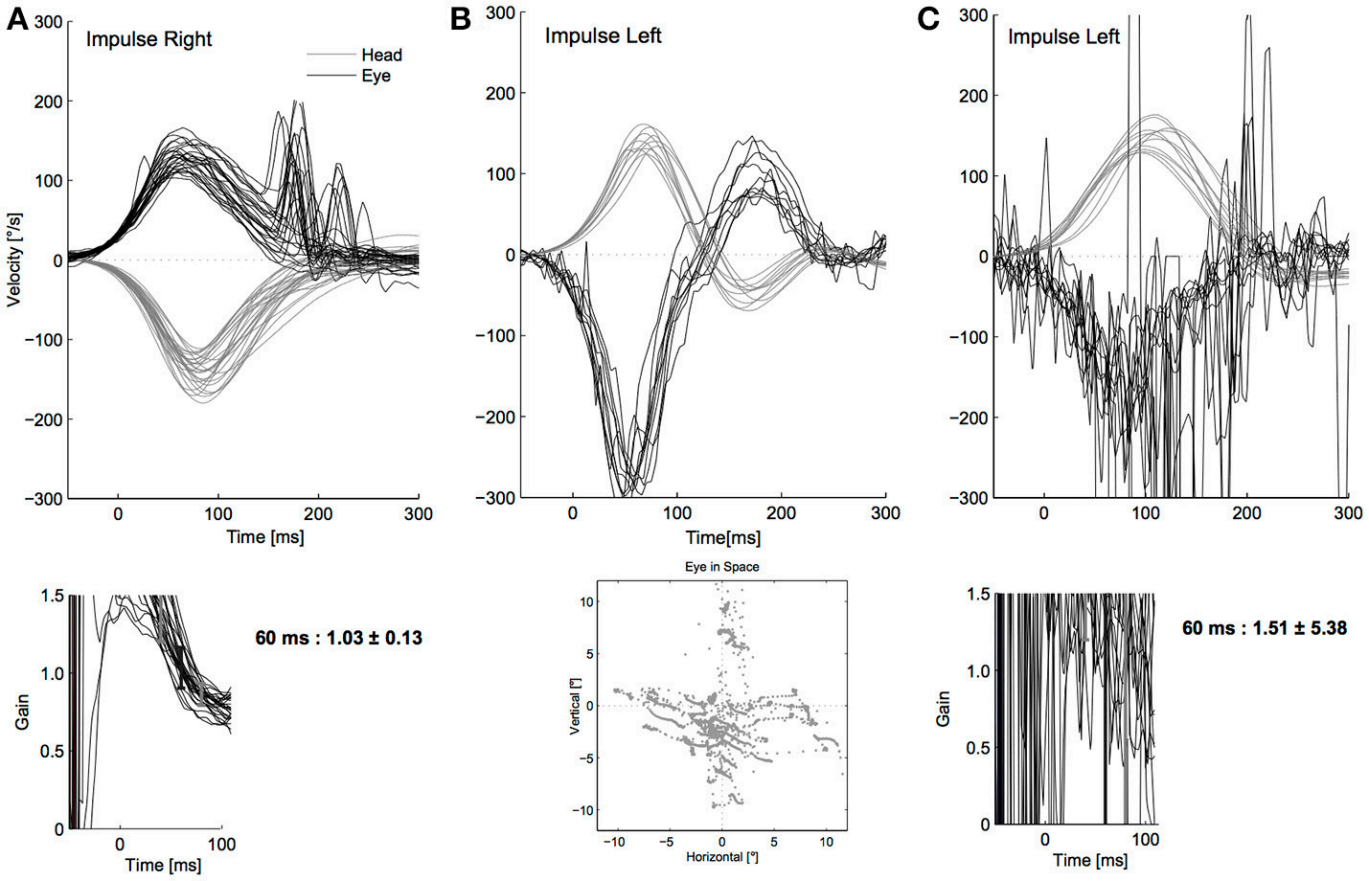
Relevant restrictive artifacts in our cohort. In this figure shows the relevant restrictive artifacts in our study: **(A)** slippage: the eye movement (top, black) precedes the head movement (top, gray). This leads to a calculated normal gain of 1.0 at 55–65 ms (see time course of the gain, bottom). A vestibulo-ocular reflex (VOR) deficit (note distinct re-fixation saccades, top) might not be detected because of the phase shift falsely increasing the gain value. **(B)** Calibration problems: due to a false calibration (bottom), the eye to head movement ratio is miscalculated resulting in an inappropriately low or high (as in this example) gain value without matching re-fixation saccades. Note the combination with the (not dominating) slippage artifact. For a correct example of calibration see Figure 1. **(C)** noise: oscillations of the eye movement trace (top, black), probably due to blinks, impaired pupil detection (e.g., narrow palpebral fissure, wearing mascara) or the examiner touching the goggles. A reliable gain calculation (bottom) is not possible. In this example, the former categories blinks, multiple VOR peaks and oscillations are present (Figure 2), summarized as “noise.”

Twenty-two of the 681 participants' vHITs (3.2%) were not interpretable due to restrictive mistakes (insufficient technical quality, examples see Figure 3). Of these, 14 (63.6%) showed slippage (Figure 3A), calibration was faulty in four (18.2%, Figure 3B), three displayed blinks and multiple peaks (13.6%, with one vHIT also displaying trace oscillations, Figure 3C), and the head stimulus was too slow in one participant (4.6%). We did not observe “wrong VOR direction” or any “unclassifiable artifacts” as restrictive mistakes. Thirteen cases of slippage showed a phase shift with eye movement preceding head movement, one case had a phase shift with head movement preceding eye movement. With blinks and multiple peaks always occurring together, and trace oscillations always being associated with these two, we grouped these three mistakes in a new category “noise” (see also Discussion and Figure **5**).

The overall rate of restrictive mistakes decreased significantly during the study, with 12% (9/75) within the first 25 tested participants by each of the examiners and 2.1% (13/605) during the rest of the examinations (two-sided test for proportion *p* < 0.0001). After the first 50 tested participants per examiner the rate of restrictive mistakes decreased to 1.3% (7/530). 72.7% (16/22) of all mistakes occurred during the first 4 months of testing. After 6 months of the study, there were no more slippage, calibration or velocity problems, but only two cases of too noisy vHIT (occurring in month 11 and 13).

**Figure 4:**
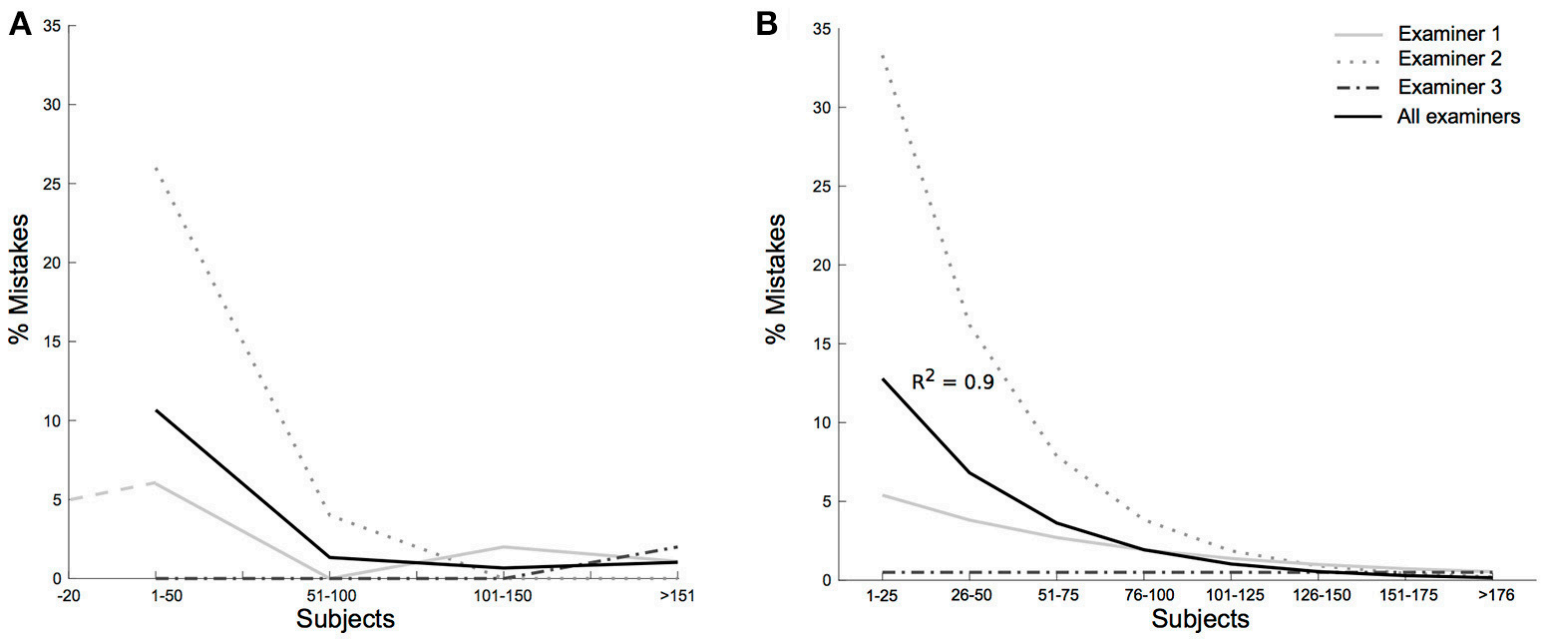
Restrictive mistakes per examiner and individual learning curves. **(A)** Shows the rate of restrictive mistakes for the individual examiners (gray lines) and the cumulative rate (black line) with participants grouped in chunks of 50. After the first 50 tested participants per examiner the rate of restrictive mistakes decreased from 10% (15/150) to 1.3% (7/530). Examiner 1 participated in a pilot study, represented in a dashed line. **(B)** Shows exponential fits for each of the three examiners. Participants were grouped in chunks of 25. The decrease of non-interpretable vHITs over the number of tested participants was estimated fitting the rate of mistakes with an exponential curve. Examiner 2 with most errors in the beginning of the study showed an *R*^2^ of 0.97 (examiner 1 *R*^2^ = 0.34, examiner 3 *R*^2^ = 0.00). The overall exponential fit for all examiners' restrictive mistakes resulted in an *R*^2^ of 0.90.

Suspecting the vHIT procedure needed some amount of personal skill and practice, we investigated its quality per examiner. The number of restrictive mistakes differed greatly between examiners (Figure 4A, *p* = 0.0007) with examiner 2 having a percentage of 6.8% of non-interpretable vHITs (15/219), examiner 1 2.3% (6/261), and examiner 3 0.5% (1/200). The relative importance of identified restrictive mistakes also differed between examiners (examiner 2: 80% slippage, 13% calibration problems, 7% noise, examiner 1: 33% calibration problems, 33% slippage, 17% noise, 17% low velocity, examiner 3: 100% noise). Figure 4B shows the exponential descent of non-interpretable vHITs of all examiners over the rising number of tested participants per examiner (exponential fit for all examiners with *R*^2^ = 0.90, examiner 2: *R*^2^ = 0.97, examiner 1: *R*^2^ = 0.34, examiner 3: *R*^2^ = 0.00).

## Discussion

### Rate of insufficient vHIT technical quality restricting interpretation (restrictive mistakes)

In our study, 3.2% of participants (22/681) had vHIT with restrictive mistakes. This number is comparable to that of two smaller studies where experts performed vHIT: Mossman et al. (13) had to discard 5% (3/63) of healthy subjects' data due to technical reasons; Mantokoudis et al. (10) analyzing 26 patients with acute vestibular syndrome found 42% of traces non-interpretable but overall, could diagnose all patients (11). With our discard rate of 3.2% applied to their patient population of 26, 0.8 patients should have been non-interpretable.

### Relevant restrictive mistakes and suggestion for avoidance

We found four relevant restrictive mistakes: slippage (63.6%), calibration problems (18.2%), noise (13.6%), and insufficient peak head velocity (4.6%). The overall rate of restrictive mistakes diminished quickly with study time, proposing a prompt learning effect by training. This puts into perspective the large number of artifacts described in the literature (9, 10, 12–16). Importantly, in our study, examiners without any prior vHIT skills achieved the low number of restrictive mistakes.

Slippage is an important restrictive mistake. Suh et al. (12) have studied slippage due to a loose goggle strap in healthy participants. Loose goggle straps lead to slippage with head motion preceding eye motion. This type of slippage was rare in our study, probably because we greatly emphasized fastening the strap in our trainings (see Supplement 1). In our study, slippage predominantly showed a phase shift with preceding eye velocity, probably due to involuntary stretching of the participant's skin or touching the goggle strap. After putting more emphasis on this slippage type during the first 6 months of study time, slippage did not occur any more. This again underlines the importance of training. We did not need to apply further measures proposed to prevent slippage such as dental paste (18).

The second most common restrictive mistake - calibration problems - did not require a specific training in the course of the study, but decreased with repetitively emphasizing the need for quality control, and if necessary, repetition.

Insufficient head stimuli were avoidable by training. We provided the examiners with leads like relaxing the participant by starting with slow head movements before the vHIT to better tolerate the high peak head velocity necessary (see Supplement 1).

Other artifacts described in the literature and caused by blinks, touching the goggles or impaired pupil detection only very rarely restricted interpretation in our study (3/681 participants). We subsumed them under the term “noise.”

**Figure 5:**
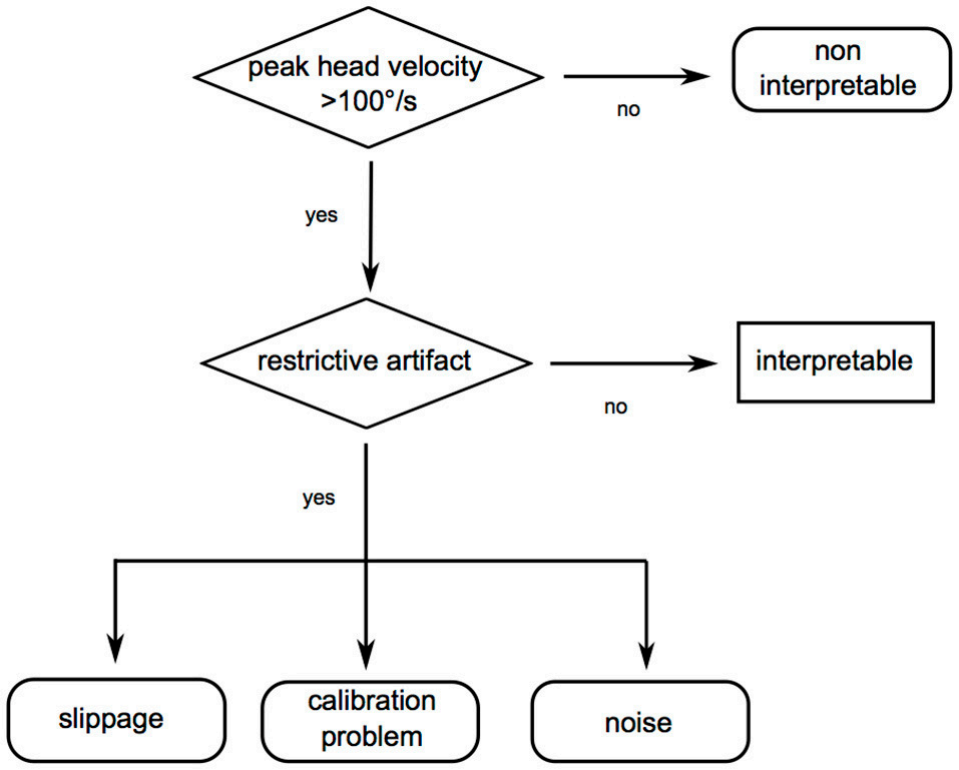
Suggested flowchart to check for video head impulse test (vHIT) quality. In our study, we could reduce the relevant restrictive mistakes to four categories (insufficient head stimulus velocity, slippage, calibration problems, and noise). As a result, we suggest a simplified flowchart to check for sufficient vHIT quality.

Overall, we could reduce the types of restrictive mistakes rendering a confident vHIT interpretation impossible to four. This facilitates vHIT training and is encouraging especially in sight of the increasing importance of vHIT for non-specialists (e.g., in the emergency room). This is supported by Mantokoudis et al. (11), who report no significant or clinically relevant effect on data-sensitivity or specificity by filtering vHITs manually for artifacts in a study of 26 patients. To facilitate scanning vHITs for restrictive mistakes we developed a simplified flowchart (Figure 5).

### Individual learning and applying the vHIT

To our knowledge, this is the first prospective study about inexperienced examiners learning vHIT. First, it was possible for all three examiners to learn applying the vHIT with a reasonable rate of restrictive mistakes within two trainings of two to 3 h. Machner et al. (14) describe a learning time period of 2 days for health personnel, in comparison to 2 weeks for electronystagmography / calorics. The inter-examiner difference in the rate of restrictive mistakes as well as in their kind, suggests that people have different “talents” for applying the vHIT. This underlines the importance of individual training.

### Study limitations

The study was conducted in 2013/2014, and currently, the analysis of six semicircular canals is becoming more popular. Most probably, the main study conclusions could be extrapolated to the examination of the vertical canals. This study was performed with one of the available vHIT devices, and the results are therefore valid for this system. Differences in methodology between vHIT systems, e.g., concerning calibration, are not reflected here.

## Conclusion

Few categories suffice to explain restrictive mistakes in vHIT testing. With goggle slippage and calibration problems being most important, trainers should emphasize the importance of tight goggles, not touching the strap or stretching the patient's skin while applying the head impulse, and of correct calibration. Although vHIT should not be considered a plug and play device, experience, and individual training quickly and effectively improve vHIT quality, leading to high usability.

## Data availability statement

All relevant data is contained within the manuscript.

## Author contributions

EG, RH, AP, ES, and NL did study conception. MH and NL performed vHIT trainings and supervision; MH, MS, ES, and NL analyzed and interpreted the data. MS, CR, MM, and RS contributed to the statistical analysis. MH and NL drafted the initial manuscript and revised the manuscript. All authors have read and approved the final manuscript.

### Conflict of interest statement

ES is general manager and a shareholder of EyeSeeTec GmbH. NL is a shareholder and paid consultant to EyeSeeTec GmbH. CR was an employee of EyeSeeTec GmbH. The remaining authors declare that the research was conducted in the absence of any commercial or financial relationships that could be construed as a potential conflict of interest.

## Abbreviations

FF4: second follow-up of the KORA S4 population-based health survey
KORA: Cooperative Health Research in the Region of Augsburg
vHIT: video head impulse test
VOR: vestibulo-ocular reflex.

## References

[1] Rambold HA. Economic management of vertigo/dizziness disease in a county hospital: video-head-impulse test vs. caloric irrigation. Eur Arch Otorhinolaryngol. (2015) 272:2621–8. 10.1007/s00405-014-3205-1 25078154

[2] van Esch BF Nobel-Hoff GE van Benthem PP van der Zaag-Loonen HJ Bruintjes TD. Determining vestibular hypofunction: start with the video-head impulse test. Eur Arch Otorhinolaryngol. (2016) 273:3733–9. 10.1007/s00405-016-4055-9 27113255

[3] Newman-Toker DE Saber Tehrani AS Mantokoudis G Pula JH Guede CI Kerber KA. Quantitative video-oculography to help diagnose stroke in acute vertigo and dizziness: toward an ECG for the eyes. Stroke (2013) 44:1158–61. 10.1161/STROKEAHA.111.000033 23463752 PMC8448203

[4] Mantokoudis G Tehrani AS Wozniak A Eibenberger K Kattah JC Guede CI. VOR gain by head impulse video-oculography differentiates acute vestibular neuritis from stroke. Otol Neurotol. (2015) 36:457–65. 10.1097/MAO.0000000000000638 25321888

[5] Bartl K Lehnen N Kohlbecher S Schneider E. Head impulse testing using video-oculography. Ann N Y Acad Sci. (2009) 1164:331–3. 10.1111/j.1749-6632.2009.03850.x 19645921

[6] MacDougall HG Weber KP McGarvie LA Halmagyi GM Curthoys IS. The video head impulse test: diagnostic accuracy in peripheral vestibulopathy. Neurology (2009) 73:1134–41. 10.1212/WNL.0b013e3181bacf85 19805730 PMC2890997

[7] Halmagyi GM Curthoys IS. A clinical sign of canal paresis. Arch Neurol. (1988) 45:737–9. 3390028 10.1001/archneur.1988.00520310043015

[8] Weber KP MacDougall HG Halmagyi GM Curthoys IS. Impulsive testing of semicircular-canal function using video-oculography. Ann N Y Acad Sci. (2009) 1164:486–91. 10.1111/j.1749-6632.2008.03730.x 19645955

[9] Agrawal Y Schubert MC Migliaccio AA Zee DS Schneider E Lehnen N. Evaluation of quantitative head impulse testing using search coils versus video-oculography in older individuals. Otol Neurotol. (2014) 35:283–8. 10.1097/MAO.0b013e3182995227 24080977 PMC4532669

[10] Mantokoudis G Saber Tehrani AS Kattah JC Eibenberger K Guede CI Zee DS. Quantifying the vestibulo-ocular reflex with video-oculography: nature and frequency of artifacts. Audiol Neurootol. (2015) 20:39–50. 10.1159/000362780 25501133

[11] Mantokoudis G Saber Tehrani AS Wozniak A Eibenberger K Kattah JC Guede CI. Impact of artifacts on VOR gain measures by video-oculography in the acute vestibular syndrome. J Vestib Res. (2016) 26:375–85. 10.3233/VES-160587 27814312 PMC6054448

[12] Suh MW Park JH Kang SI Lim JH Park MK Kwon SK. Effect of goggle slippage on the video head impulse test outcome and its mechanisms. Otol Neurotol. (2017) 38:102–9. 10.1097/MAO.0000000000001233 27956722

[13] Mossman B Mossman S Purdie G Schneider E. Age dependent normal horizontal VOR gain of head impulse test as measured with video-oculography. J Otolaryngol Head Neck Surg. (2015) 44:29. 10.1186/s40463-015-0081-7 26141721 PMC4506627

[14] Machner B Sprenger A Fullgraf H Trillenberg P Helmchen C. Video-based head impulse test. Importance for routine diagnostics of patients with vertigo. Nervenarzt (2013) 84:975–83. 10.1007/s00115-013-3824-6 23839059

[15] Halmagyi GM Chen L MacDougall HG Weber KP McGarvie LA Curthoys IS. The video head impulse test. Front Neurol. (2017) 8:258. 10.3389/fneur.2017.00258 28649224 PMC5465266

[16] Blodow A Helbig R Wichmann N Bloching M Walther LE. The video head impulse test: first clinical experiences. HNO (2013) 61:327–34. 10.1007/s00106-012-2592-0 23588677

[17] Heuberger M Saglam M Todd NS Jahn K Schneider E Lehnen N. Covert anti-compensatory quick eye movements during head impulses. PLoS ONE (2014) 9:e93086. 10.1371/journal.pone.0093086 24732783 PMC3986070

[18] Versino M Colagiorgio P Sacco S Colnaghi S Ramat S. Artifact avoidance for head impulse testing. Clin Neurophysiol. (2014) 125:1071–3. 10.1016/j.clinph.2013.09.024 24128790

