# Supplementary material for: Usability of the Video Head Impulse Test: Lessons From the Population-Based Prospective KORA Study
Source: Front Neurol. 2018 Aug 17;9:659. doi: 10.3389/fneur.2018.00659 (PMC6107793; doi:10.3389/fneur.2018.00659)
Supplement: Supplementary file 1 [file Data_Sheet_1.PDF]

Supplementary Material

Usability of the video head impulse test: lessons from the population-based prospective KORA study

Maria Heuberger[1,2,*], Eva Grill[2,3,4], Murat Sağlam[2], Cecilia Ramaioli[2,8], Martin Müller[5], Ralf Strobl[2,3], Rolf Holle[6], Annette Peters[7], Erich Schneider[8], Nadine Lehnen[2,8,9]

[1] Department of Neurology, University Hospital Munich, Ludwig-Maximilians Universität München, Munich, Germany
[2] German Center for Vertigo and Balance Disorders, University Hospital Munich, Ludwig-Maximilians Universität München, Munich, Germany
[3] Institute for Medical Information Processing, Biometry and Epidemiology, Faculty of Medicine, Ludwig-Maximilians Universität München, Munich, Germany
[4] Munich Center of Health Sciences, Ludwig-Maximilians Universität München, Munich, Germany
[5] Faculty of Applied Health and Social Sciences, Rosenheim University of Applied Sciences, Rosenheim, Germany
[6] Institute of Health Economics and Health Care Management, Helmholtz Zentrum München, German Research Center for Environmental Health, Neuherberg, Germany
[7] Institute of Epidemiology, Helmholtz Zentrum München, German Research Center for Environmental Health, Neuherberg, Germany
[8] Institute of Medical Technology, Brandenburg University of Technology, Cottbus-Senftenberg, Germany
[9] Department of Psychosomatic Medicine and Psychotherapy, Klinikum rechts der Isar, Technical University of Munich, Munich, Germany

*** Correspondence:**
Maria Heuberger
Maria.Heuberger@med.uni-muenchen.de

Supplement 1: Standard Operating Procedure for video head impulse testing.

1 Background

The video head impulse test (vHIT) is a routine examination in the work-up for dizziness and vertigo. The vHIT examines the vestibulo-ocular reflex (VOR) which stabilizes gaze during passive head movement. The examiner turns the participant's head quickly, i.e., with high acceleration, but with small amplitude, while participants fixate a point straight ahead. If the semicircular canal is intact, the participant is able to fixate the point during head movement (figure 1A). In case of a vestibular deficit, eye movement follows head movement until a re-fixation saccade brings gaze back to the target (figure 1B). Eye movement is recorded by a camera (video-oculography), head movement with inertial sensors. The VOR gain (median of ratios of eye and head velocity in a 10ms window between 55ms and 65ms after the head movement starts) is calculated as an objective value for semicircular canal

function. Normal gain values are around one as the eye movement completely compensates for the head movement.

2 Contraindications

Exclusion criteria are problems of the cervical spine, e.g. an acute cervical disc herniation or spinal fracture. To investigate this, participants will be asked if they have any known problems of the cervical spine or acute neck pain.

3 Testing procedure

3.1 Requirements

3.1.1 Material: EyeSeeCam system, fixation point

3.1.2 Participant:

- Whenever possible, dark mascara should be removed, because it can interfere with pupil detection.
- Glasses have to be removed; whenever possible, contact lenses should be worn.
- The camera normally assesses movement of the left eye, if necessary (e.g. in the case of a glass eye), it can be changed to the right side.
- Hearing aids should be removed to prevent interferences with the goggle strap.

3.1.3 Testing conditions:

- The participant should be seated two meters from a wall, facing the wall (the chair position is marked on the floor).
- The room light should be switched on to ensure equal lighting conditions.
- The participant's head should be in a 25° head-down position.
- The fixation point should be positioned at participant' eye level.

3.2 Preparation

- Position the camera cable centered in the back of the head. This is important to avoid touching it during head impulses.
- Fasten the goggle strap. This is essential to avoid the artifact "slippage", e.g. a delayed recording of the eye movement in relation to the head movement.
- Optimize pupil detection by focusing and adjusting the camera. The pupil should be centered. To avoid partial occlusion by receding eyelids, participants can be told to open their eyes wide, the eyelids can be torn gently under the rim of the goggles.

3.3 Calibration

- Ask the participant if all five appearing laser points are clearly visible. If not, special lenses can be applied to the goggles for short sight correction. If this does not help, the participant can be moved closer to the wall (1m).
- Instruct participants to fixate the announced point without moving their head (middle - right - left - middle - up - down - middle - right - left - middle - up - down - middle).

- Evaluate the calibration: a successful calibration should form a cross with accumulations in the instructed laser points (figure 1C). A false calibration shows scattered gaze points and has to be repeated.

3.4 Applying head impulses

- Instruct the participant to look at the fixation point, to try not to blink during the head movement, and to try not to resist the head movement. Instructing the participant to clench his or her teeth may help with the application of high acceleration head motion.
- Place both hands on the jaw of the participant from behind. The head of the participant (25° head-down position) should be turned horizontally to one side with high acceleration and small amplitude (velocity 150-250°/s, amplitude 6-12°, 10-15 HITs to each side). The direction should not be predictable for the participant and there should be different time intervals between the head impulses. The head should be held in the end position for a few seconds. Peak head velocity should be assessed right after each head impulse. If necessary, try relaxing the participant with slow head movements before starting the vHIT for a better tolerance of the high peak head velocity necessary. It is important to avoid touching the goggles or stretching the participant’s skin while applying the head impulse to avoid artifacts.
